# Machine Learning for Preoperative Assessment and Postoperative Prediction in Cervical Cancer: Multicenter Retrospective Model Integrating MRI and Clinicopathological Data

**DOI:** 10.2196/69057

**Published:** 2025-09-12

**Authors:** Shuqi Li, Chenyan Guo, Yufei Fang, Junjun Qiu, He Zhang, Lei Ling, Jie Xu, Xinwei Peng, Chuchu Jiang, Jue Wang, Keqin Hua

**Affiliations:** 1 Shanghai Key Lab of Female Reproductive Endocrine Related Diseases, Shanghai Key Lab of Reproduction and Development, Obstetrics and Gynecology Hospital of Fudan University, 218 Shenyang Road, Shanghai, 200433, China, 86 021 33189900; 2 Department of Pharmaceutical Sciences, Academy of Pharmacy, Xi’an Jiaotong Liverpool University, Suzhou, China; 3 Shanghai Artificial Intelligence Laboratory, Shanghai, China

**Keywords:** cervical cancer, integrated prediction model, machine learning, multimodal integration, diagnostic model, prognosis prediction model

## Abstract

**Background:**

Machine learning (ML) has been increasingly applied to cervical cancer (CC) research. However, few studies have combined both clinical parameters and imaging data. At the same time, there remains an urgent need for more robust and accurate preoperative assessment of parametrial invasion and lymph node metastasis, as well as postoperative prognosis prediction.

**Objective:**

The objective of this study is to develop an integrated ML model combining clinicopathological variables and magnetic resonance image features for (1) preoperative parametrial invasion and lymph node metastasis detection and (2) postoperative recurrence and survival prediction.

**Methods:**

Retrospective data from 250 patients with CC (2014‐2022; 2 tertiary hospitals) were analyzed. Variables were assessed for their predictive value regarding parametrial invasion, lymph node metastasis, survival, and recurrence using 7 ML models: K-nearest neighbor (KNN), support vector machine, decision tree, random forest (RF), balanced RF, weighted DT, and weighted KNN. Performance was assessed via 5-fold cross-validation using accuracy, sensitivity, specificity, precision, F1-score, and area under the receiver operating characteristic curve (AUC). The optimal models were deployed in an artificial intelligence–assisted contouring and prognosis prediction system.

**Results:**

Among 250 women, there were 11 deaths and 24 recurrences. (1) For preoperative evaluation, the integrated model using balanced RF achieved optimal performance (sensitivity 0.81, specificity 0.85) for parametrial invasion, while weighted KNN achieved the best performance for lymph node metastasis (sensitivity 0.98, AUC 0.72). (2) For postoperative prognosis, weighted KNN also demonstrated high accuracy for recurrence (accuracy 0.94, AUC 0.86) and mortality (accuracy 0.97, AUC 0.77), with relatively balanced sensitivity of 0.80 and 0.33, respectively. (3) An artificial intelligence–assisted contouring and prognosis prediction system was developed to support preoperative evaluation and postoperative prognosis prediction.

**Conclusions:**

The integration of clinical data and magnetic resonance images provides enhanced diagnostic capability to preoperatively detect parametrial invasion and lymph node metastasis detection and prognostic capability to predict recurrence and mortality for CC, facilitating personalized, precise treatment strategies.

## Introduction

As the fourth leading cause of cancer-related death in women, cervical cancer (CC) accounted for approximately 661,000 new cases and 341,800 deaths worldwide in 2022 [1]. Despite advances in clinical management, up to 30% of patients continue to succumb to the disease, resulting in a disproportionately high global burden [2]. However, current methods for preoperative evaluation and postoperative prognosis prediction in patients with CC remain insufficiently comprehensive. Preoperative assessment relies heavily on pelvic magnetic resonance (MR) imaging to identify primary lesions [3], whereas the recognition rates for parametrial invasion and lymphatic metastases remain inconsistent [4]. Furthermore, for postoperative prognosis prediction, the International Federation of Gynecology and Obstetrics (FIGO) staging system is currently accepted as the clinical standard. However, it fails to fully account for patient heterogeneity, including factors such as age, general health status, and tumor markers. Therefore, a personalized prognostic estimation system is urgently needed. To address this, researchers have explored various statistical methods—such as logistic regression and Cox proportional hazards models—to estimate survival and recurrence outcomes on an individual basis [5,6]. Nonetheless, traditional statistical models are often limited in their ability to handle large, complex datasets and make accurate predictions in dynamic clinical environments. To this end, a more accurate and personalized prediction model—incorporating both preoperative and postoperative evaluation—is urgently needed to optimize treatment decisions and follow-up strategies for patients with CC.

In recent years, machine learning (ML)—which involves the development of dynamic algorithms capable of making data-driven decisions—has emerged as a novel method for processing medical data and has been widely applied to various diseases [7]. In the field of CC, our previous multicenter study developed a web-based calculator to predict prognosis in 5112 patients with CC using various ML models, which demonstrated better predictive accuracy than traditional statistical models [8]. However, similar to other studies [9-11], only clinicopathological information was included in the development of this ML model. Notably, with technological advancements, medical imaging can now reveal information imperceptible to the naked eye—even for experienced clinicians. Consequently, an increasing number of studies have used deep learning (DL) algorithms on MR images for the diagnosis [12,13] and classification [14] of CC. However, most existing studies have focused on lesion identification [15,16] and radiotherapy response prediction [17,18]. Currently, there is a lack of ML models that integrate both clinical and imaging data to predict prognosis in patients with CC. Therefore, we aimed to develop an integrated ML model that uses both clinical and imaging data to enhance preoperative evaluation and postoperative prognosis prediction.

Specifically, the model is designed to (1) accurately evaluate parametrial involvement and lymph node metastasis on pelvic MR images prior to surgery to better inform surgical planning, and (2) predict individualized postoperative recurrence and survival outcomes to support precise, personalized adjuvant treatment and follow-up strategies.

## Methods

### Ethical Considerations

This retrospective multicenter cohort study received ethical approval from the Institutional Review Board of the Obstetrics and Gynecology Hospital of Fudan University (Shanghai, China; approval no. 2019‐87) and registered with the Chinese Clinical Trial Registry (ChiCTR1900028702). Oral informed consent was obtained from all participants via telephone follow-up in accordance with institutional ethical standards. All personally identifiable information was permanently removed prior to analysis. Data were coded using unique identifiers and stored securely on encrypted, password-protected servers with role-based access controls to ensure confidentiality. No compensation was provided to participants.

### Patients

**Figure 1.**
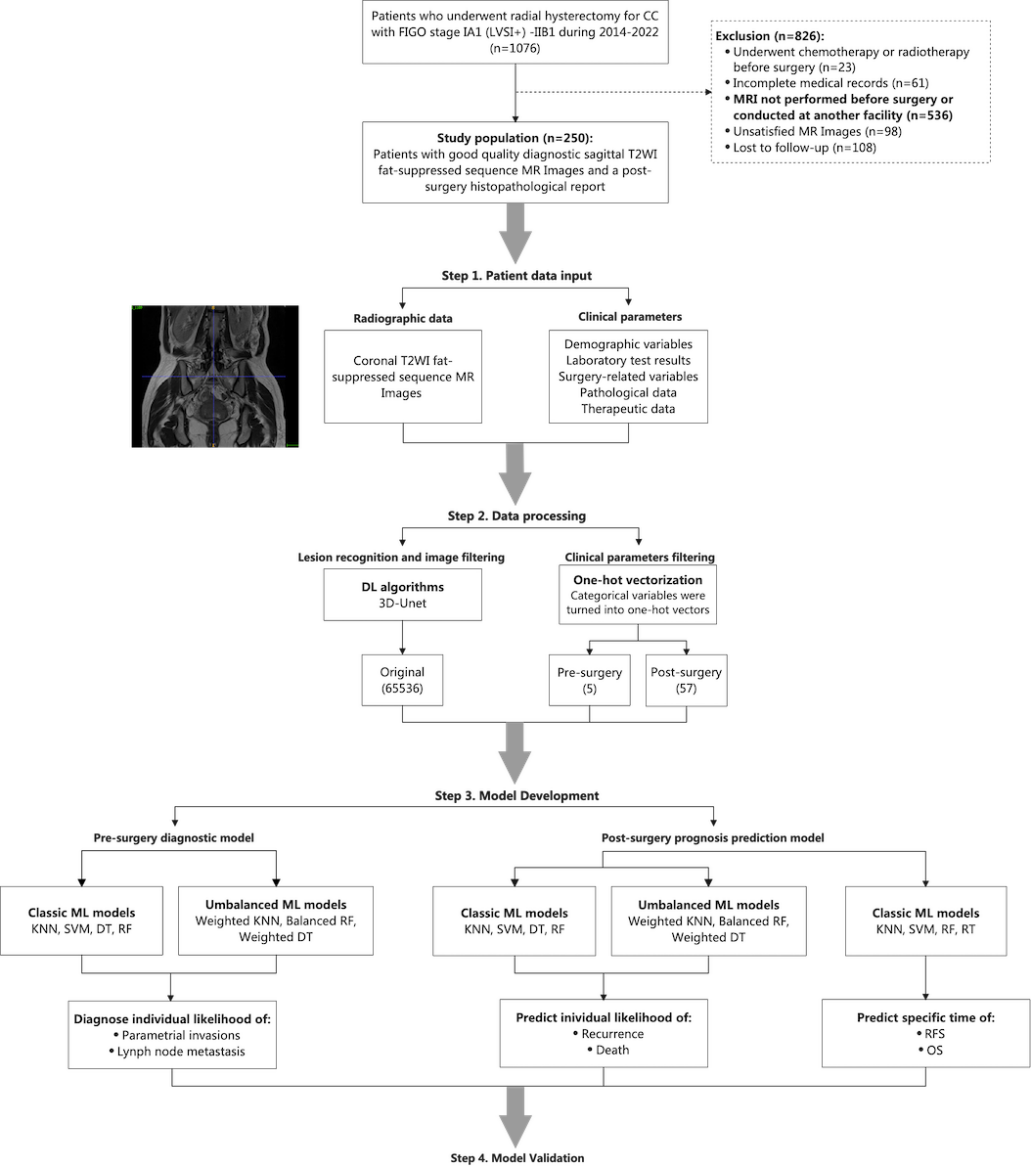
Flow diagram illustrating the selection process for the study population and the sequential stages of machine learning model development. FIGO: International Federation of Gynecology and Obstetrics; LVSI: lymph-vascular space invasion; T2WI: T2-weighted imaging; MR: magnetic resonance; KNN: K-nearest neighbor; SVM: support vector machine; DT: decision tree; RF: random forest; RT: regression tree; RFS: recurrence-free survival; OS: overall survival.

A total of 1076 patients with CC who underwent surgical resection between January 2014 and December 2022 were identified from 2 tertiary hospitals in China: the Obstetrics and Gynecology Hospital affiliated with Fudan University and Shanghai First Maternity and Infant Hospital. The inclusion criteria were as follows: (1) pathologically confirmed FIGO stage IA1 with positive lymph-vascular space invasion (LVSI) to stage IIB CC; (2) radical hysterectomy performed according to National Comprehensive Cancer Network (NCCN) guidelines appropriate to the disease stage at the time [19-21]; (3) availability of high-quality preoperative pelvic contrast-enhanced MR images, including the coronal T2-weighted imaging (T2WI) fat-suppressed sequence; and (4) at least 3 years of follow-up data. The exclusion criteria were as follows: (1) receipt of chemotherapy or radiotherapy before surgery (n=23); (2) incomplete medical records (n=61); (3) absence of preoperative MR imaging (MRI) or MRI performed at another institution (n=536); (4) unsatisfactory MR image quality (n=98); and (5) loss to follow-up (n=108). The final study population comprised 250 patients (Figure 1).

### Clinical Information

For eligible patients, demographic data, laboratory test results, treatment details, and tumor characteristics were collected from medical records. All records were reviewed concurrently by 3 experts and independently verified for accuracy by 2 additional reviewers. Following diagnosis, demographic variables—including age and comorbidity (hypertension or diabetes)—as well as laboratory data, including squamous cell carcinoma antigen levels and human papillomavirus (HPV) infection status, were recorded. The history of the loop electrosurgical excision procedure was also noted.

All surgical procedures during the study period were performed by faculty members with completed fellowship training in gynecologic oncology. Surgical data included surgical approach, operative time, estimated blood loss, and use of blood transfusion. Tumor characteristics included FIGO stage, tumor size, histologic type, depth of stromal invasion (DSI), LVSI, surgical margin status, parametrial involvement, lymph node metastasis, keratinization, degree of differentiation, and expression of P53, P16, and Ki-67. According to the NCCN guidelines [3], all patients received adjuvant therapy if they met one of the following criteria: (1) presence of any high-risk factor, including positive surgical margins, parametrial involvement, or lymph node metastasis; or (2) fulfillment of any Sedlis criteria [22] for intermediate-risk factors, including tumor size, LVSI, and DSI.

Patients were followed according to the 2022 NCCN guidelines [3] after discharge and completion of initial treatment. HPV testing, liquid-based cytology, tumor marker evaluation, and ultrasonography were conducted every 3 months for the first 2 years, every 6 months for the next 2 years, and annually thereafter. Chest computed tomography (CT), contrast-enhanced upper abdominal CT, and pelvic MRI were performed annually. Telephone follow-ups were also conducted, and patients with symptoms or abnormal findings suggestive of recurrence were advised to undergo the aforementioned tests. In cases of suspected organ or lymph node metastasis, needle aspiration biopsy was performed when clinically indicated.

### Region of Interest Delineation on MR Images

Coronal preoperative T2WI fat-suppressed pelvic contrast-enhanced MR images were collected for all 250 patients. This included both the original MR sequence source images and lesion segmentation images annotated by radiologists. The MR source images were first normalized before further processing. To standardize the 2 types of MR source image resolutions (256×256 pixels and 320×320 pixels), images of 320×320 pixels were center-cropped to 256×256 pixels. Meanwhile, the pixel intensity values, which originally ranged from 0 to over 1000, were rescaled to the range [0, 1]. After obtaining standardized MR source images, 2 experienced radiologists performed lesion annotations. The region of interest for CC was manually delineated on T2WI images using ITK-SNAP (version 3.8.0). Each radiologist delineated half of the cases and independently reviewed the other half. In cases of disagreement, the final decision was reached through discussion or by consulting a third radiologist. During the segmentation process, the radiologists were blinded to the patients’ clinical information.

After obtaining standardized MR source images, 2 experienced radiologists performed lesion annotations. The region of interest for CC was manually delineated on T2WI images using ITK-SNAP (version 3.8.0). Each radiologist delineated half of the cases and independently reviewed the other half. In cases of disagreement, the final decision was reached through discussion or by consulting a third radiologist. During the segmentation process, the radiologists were blinded to the patients’ clinical information.

Subsequently, cervical lesion identification was modeled using the radiologist-annotated MR images. An optimized algorithm based on the 3D U-Net architecture [23], embedded with a squeeze-and-excitation layer [24], was adopted to segment the lesion pixels. Following multiple preprocessing steps (see Supplementary Materials in Multimedia Appendix 1), the input data (C×W×H×D) were compressed and normalized using the Sigmoid activation function [25], then reshaped to their original dimensions. The grayscale output was segmented by computing the probability of each pixel being classified as a positive sample through the Sigmoid function. To address the issue of sample imbalance—where MR image sequences contained significantly more frames with lesion markers than without—an improved Focal Loss function [26] was implemented. A weighting factor (α) was applied to balance positive and negative samples, while a power function was used to reduce the loss contribution of easy samples. The optimal parameters used in this study were *α*=.25 and *γ*=2.

### Outcome

Parametrial invasion and lymphatic metastasis were 2 key pathological indicators derived from the surgical specimens of cervical carcinoma. Parametrial invasion refers to the infiltration of neoplastic cells beyond the cervix into the surrounding connective tissue (parametrium), while lymphatic metastasis denotes the spread of malignant cells to regional lymph nodes.

For survival outcomes, recurrence-free survival (RFS) was defined as the interval from the initial CC diagnosis to either the first documented recurrence or the last follow-up. Overall survival (OS) was defined as the interval from diagnosis to CC–related death or the last follow-up. Regarding recurrence classification, local recurrence was defined as the pathologically confirmed first reappearance of cancer in the cervix or vagina following complete treatment, confined to the pelvic region. Distant recurrence was defined as the first pathologically confirmed relapse beyond the pelvis, including peritoneal dissemination or metastasis to distant organs.

### Dataset Processing

After selecting MR source images that corresponded to lesion-annotated segmentation files, the filtered original images (256×256 pixels) were flattened into 65,536-dimensional vectors. The clinical dataset consisted of 22 variables (Table S1 in Multimedia Appendix 1), including both continuous and categorical features. Continuous variables were normalized using min-max scaling, rescaling each feature into the [0, 1] range using the following formula:


$$X_{norm}=\frac{X-X_{min}}{X_{max}-X_{min}}$$

where $X_{min}$ and $X_{max}$ represent the minimum and maximum observed values of that feature. For example, for a variable ranging from 2 to 10, a value of 5 would be transformed as: (5–2)/(10–2)=0.375. Categorical variables were encoded using one-hot encoding, which converts nominal variables into orthogonal binary vectors. For a categorical feature with 3 classes (1, 2, 3), the transformation was as follows: Class 1 → [1, 0, 0]; Class 2 → [0, 1, 0]; and Class 3 → [0, 0, 1].

After preprocessing, categorical and continuous variables were concatenated into 5-dimensional (preoperative) and 57-dimensional (postoperative) clinical feature vectors. These clinical vectors were then fused with the 65,536-dimensional image vectors, resulting in integrated feature vectors of 65,593 dimensions.

### Establishment of Integrated Models

Two integrated models were developed: (1) The preoperative recognition model integrated preoperative clinical parameters (age, comorbidity, HPV status, squamous cell antigen carcinoma level, and loop electrosurgical excision procedure history) and MR images to generate binary classifications for parametrial invasion and lymph node metastasis; and (2) the postoperative prognostic model combined 21 postoperative clinical parameters (Table S1 in Multimedia Appendix 1) and MR images to predict recurrence, mortality, and individualized RFS or OS times.

A total of 7 ML algorithms were used for model construction. These included classical ML algorithms such as K-nearest neighbor (KNN), support vector machine (SVM), decision tree (DT), and random forest (RF), as well as their imbalanced-data variants—weighted KNN, balanced RF, and weighted DT. Weighted KNN adjusted sample weights inversely proportional to class frequency. Specifically, minority class samples (in this study, positive cases) were assigned higher weights in distance calculations, forcing the algorithm to prioritize minority class neighborhoods during prediction. Balanced RF used under-sampling by randomly removing majority class samples (negative cases) in each bootstrap iteration to create balanced subsets for training individual trees. Weighted DT modified the splitting criterion by incorporating a class-weighted penalty: a weight of misclassifying a positive sample was set to amplify the cost of false negatives during node splitting. Besides, to mitigate the influence of class imbalance during model development, negative samples were down-sampled to achieve a 1:1 ratio with positive cases during training.

### Validation, Evaluation, and Implementation

The study process consisted of 4 main stages: patient data input, data preprocessing, model development, and model evaluation (Figure 1). Stratified random sampling was used to divide the complete dataset from the 2 hospitals into training and test sets at a ratio of 8:2. Model training was conducted using the training set, while final validation was performed on the test set. To minimize overfitting and reduce bias, 5-fold cross-validation was used on the training set for hyperparameter tuning.

Model performance for classification tasks was evaluated using sensitivity, specificity, accuracy, precision, F1-score, weighted accuracy, and area under the receiver operating characteristic curve (AUC). The mean absolute error (MAE) and concordance index (C-index) were used to assess the accuracy of individual-specific RFS and OS time predictions.

To facilitate clinical application and enhance accessibility for physicians, a web-based predictive diagnostic support tool was developed using Python, enabling DICOM upload and automated prediction.

### Statistical Analysis

Continuous variables were reported as means with standard deviations (SDs) for normally distributed data and as medians with interquartile ranges (IQRs) for non-normally distributed data, while categorical variables were summarized as counts and percentages. No significant interactions were observed among variables based on correlation matrix analysis. Concordance of continuous variables was assessed using intraclass correlation coefficients to evaluate consistency between pathology and imaging reports. Categorical variables were analyzed using the *χ^2^* test and the Kappa coefficient.

All statistical analyses were performed using R statistical software (version 4.1.1; R Foundation for Statistical Computing) and Python programming software (version 3.10.4; Python Software Foundation). All tests were 2-sided, and a *P* value of less than .05 was considered statistically significant unless otherwise stated.

## Results

### Baseline Characteristics of 250 Patients with Early-Stage CC

A total of 250 patients with FIGO stage IA1 (LVSI+) to IIB CC who underwent radical hysterectomy between 2014 and 2022 were included in the study population (Table 1). The median age was 48.8 years. Most patients were at stage I (n=182, 72.8%) and had a squamous histologic type (n=200, 80%). More than half of the patients (n=191, 76.4%) received adjuvant therapy. In total, 24 women (9.6%) experienced recurrence, and 11 (4.4%) died during the follow-up period. Among the recurrence cases, 9 (37.5%) were local recurrences and 15 (62.5%) were distant. Among the distant recurrences, 4 (16.7%) occurred in the thoracic region, 5 (20.8%) in the abdominal region, and 6 (25%) in bone. The median RFS was 33.8 (IQR 24.9‐42.4) months, and the median OS was 34.6 (IQR 26.0‐42.8) months. The 1-year RFS and OS rates were 94.4% and 99.2%, respectively, while the 3-year RFS and OS rates were 90.1% and 95.4%, respectively (Figure S1 in Multimedia Appendix 1).

**Table 1.** Baseline characteristics of patients with stage IA1 (LVSI+)^a^ to IIB CC^b^. Data are reported as number of patients and percentage of total in parentheses, unless otherwise noted.

Characteristics	Overall (n=250)	Train (n=200)	Test (n=50)	*P* value
Clinical variables				
Age at diagnosis (years), mean (SD^c^)	48.8 (9.6)	48.3 (9.7)	50.9 (9.1)	.09
FIGO^d^ stage, n (%)				.40
IA1	2 (0.8)	1 (0.5)	1 (2.0)	
IA2	0 (0.0)	0 (0.0)	0 (0.0)	
IB1	148 (59.2)	123 (61.5)	25 (50.0)	
IB2	32 (12.8)	26 (13.0)	6 (12.0)	
IIA1	52 (20.8)	37 (18.5)	15 (30.0)	
IIA2	14 (5.6)	11 (5.5)	3 (6.0)	
IIB	2 (0.8)	2 (1.0)	0 (0.0)	
Comorbidity, n (%)				.73
Yes	41 (16.4)	32 (16.0)	9 (18.0)	
No	209 (83.6)	168 (84.0)	41 (82.0)	
HPV^e^ infection, n (%)				.34
Yes	117 (46.8)	91 (45.5)	26 (52.0)	
HPV 16/18	74 (29.6)	60 (30.0)	14 (28.0)	
Other HPV type	43 (17.2)	31 (15.5)	12 (24.0)	
No	6 (2.4)	6 (3.0)	0 (0.0)	
Unknown	127 (50.8)	103 (51.5)	24 (48.0)	
SCCA^f^ (ng/mL), median (IQR)^g^	2.1 (1.0‐4.9)	2.1 (1.0‐5.0)	1.5 (0.8‐4.5)	.93
Post-surgery adjuvant therapy, n (%)				.94
Yes	191 (76.4)	153 (76.5)	38 (76.0)	
No	59 (23.6)	47 (23.5)	12 (24.0)	
Surgery-related variables				
Surgery approach, n (%)				
MH^h^	246 (98.4)	197 (98.5)	49 (98.0)	.56
LH^i^	221 (88.4)	175 (87.5)	46 (92.0)	
Robotic	25 (10.0)	22 (11.0)	3 (6.0)	
OH^j^	4 (1.6)	3 (1.5)	1 (2.0)	
Operative time (min), median (IQR)	180.0 (159.0‐225.0)	180.0 (157.0‐222.5)	180.0 (160.0‐230.0)	.80
Blood loss (mL), median (IQR)	200.0 (100.0‐300.0)	200.0 (100.0‐200.0)	200.0 (100.0‐300.0)	.12
Transfusion, n (%)				.26
Yes	5 (2.0)	3 (1.5)	2 (4.0)	
No	245 (98.0)	197 (98.5)	48 (96.0)	
Pathologic variables				
Tumor size (cm) , n (%)				.11
[0, 0.5)	8 (3.2)	6 (3.0)	2 (4.0)	
[0.5, 1)	0 (0.0)	0 (0.0)	0 (0.0)	
[1, 1.5)	13 (5.2)	6 (3.0)	7 (14.0)	
[1.5, 2)	13 (5.2)	12 (6.0)	1 (2.0)	
[2, 2.5)	33 (13.2)	26 (13.0)	7 (14.0)	
[2.5, 3)	29 (11.6)	26 (13.0)	3 (6.0)	
[3, 3.5)	38 (15.2)	31 (15.5)	7 (14.0)	
[3.5, 4)	39 (15.6)	31 (15.5)	8 (16.0)	
[4, 4.5)	23 (9.2)	20 (10.0)	3 (6.0)	
[4.5, 5)	23 (9.2)	16 (8.0)	7 (14.0)	
≥5	28 (11.2)	23 (11.5)	5 (10.0)	
Histology, n (%)				.20
SCC^k^	200 (80.0)	161 (80.5)	39 (78.0)	
AC^l^	29 (11.6)	25 (12.5)	4 (8.0)	
AS^m^	19 (7.6)	12 (6.0)	7 (14.0)	
Rare type	2 (0.8)	2 (1.0)	0 (0.0)	
DSI^n^, n (%)				.51
Negative	24 (9.6)	21 (10.5)	3 (6.0)	
Inner 1/3	30 (12.0)	26 (13.0)	4 (8.0)	
Middle 1/3	28 (11.2)	21 (10.5)	7 (14.0)	
Outer 1/3	168 (67.2)	132 (66.0)	36 (72.0)	
LVSI, n (%)				.20
Yes	145 (58.0)	120 (60.0)	25 (50.0)	
No	105 (42.0)	80 (40.0)	25 (50.0)	
Surgical margin involvement, n (%)				.78
Yes	32 (12.8)	25 (12.5)	7 (14.0)	
No	218 (87.2)	175 (87.5)	43 (86.0)	
Parametrial involvement, n (%)				.61
Yes	16 (6.4)	12 (6.0)	4 (8.0)	
No	234 (93.6)	188 (94.0)	46 (92.0)	
Lymph node metastasis, n (%)				.35
Yes	64 (25.6)	51 (25.5)	13 (26.0)	
Pelvic lymph nodes	54 (21.6)	45 (22.5)	9 (18.0)	
Common iliac lymph nodes	6 (2.4)	4 (2.0)	2 (4.0)	
Para-aortic lymph nodes	4 (1.6)	2 (1.0)	2 (4.0)	
No	186 (74.4)	149 (74.5)	37 (74.0)	
Keratinization, n (%)				.96
Yes	87 (34.8)	71 (35.5)	16 (32.0)	
No	92 (36.8)	73 (36.5)	19 (38.0)	
Non-SCC	50 (20.0)	39 (19.5)	11 (22.0)	
Unknown	21 (8.4)	17 (8.5)	4 (8.0)	
Differentiation, n (%)				.04
Low	1 (0.4)	1 (0.5)	0 (0.0)	
Intermediate	4 (1.6)	3 (1.5)	1 (2.0)	
High	2 (0.8)	0 (0.0)	2 (4.0)	
Unknown	243 (97.2)	196 (98.0)	47 (94.0)	
P53, n (%)				.48
–	73 (29.2)	60 (30.0)	13 (26.0)	
+	172 (68.8)	137 (68.5)	35 (70.0)	
++	0 (0.0)	0 (0.0)	0 (0.0)	
+++	0 (0.0)	0 (0.0)	0 (0.0)	
++++	0 (0.0)	0 (0.0)	0 (0.0)	
Unknown	5 (2.0)	3 (1.5)	2 (4.0)	
P16, n (%)				.27
Negative	5 (2.0)	3 (1.5)	2 (4.0)	
Positive	240 (96.0)	194 (97.0)	46 (92.0)	
Unknown	5 (2.0)	3 (1.5)	2 (4.0)	
Ki67, n (%)				.86
—	0 (0.0)	0 (0.0)	0 (0.0)	
0%‐20%	16 (6.4)	13 (6.5)	3 (6.0)	
20%‐40%	48 (19.2)	37 (18.5)	11 (22.0)	
40%‐60%	77 (30.8)	61 (30.5)	16 (32.0)	
60%‐80%	71 (28.4)	60 (30.0)	11 (22.0)	
80%‐100%	32 (12.8)	25 (12.5)	7 (14.0)	
Unknown	6 (2.4)	4 (2.0)	2 (4.0)	
Survival outcomes				
Recurrence, n (%)				.59
Yes	24 (9.6)	18 (9.0)	6 (12.0)	
Local region	9 (3.6)	7 (3.5)	2 (4.0)	
Thoracic region	4 (1.6)	2 (1.0)	2 (4.0)	
Abdominal region	5 (2.0)	3 (1.5)	2 (4.0)	
Bone	6 (2.4)	6 (3.0)	0 (0.0)	
Other regions	0 (0.0)	0 (0.0)	0 (0.0)	
No	226 (90.4)	182 (91.0)	44 (88.0)	
RFS^o^ (month), median (IQR)	33.8 (24.9‐42.4)	33.2 (24.5‐42.6)	35.0 (26.8‐41.4)	.49
Death, n (%)				.54
Yes	11 (4.4)	8 (4.0)	3 (6.0)	
No	239 (95.6)	192 (96.0)	47 (94.0)	
OS^p^ (month), median (IQR)	34.6 (26.0‐42.8)	34.2 (25.3‐43.0)	35.0 (27.9‐41.4)	.47

a LVSI: lymphovascular space invasion.

b CC: cervical cancer.

c SD: standard deviation.

d FIGO: International Federation of Gynecology and Obstetrics.

e HPV: human papillomavirus.

f SCCA: squamous cell carcinoma antigen.

g IQR: interquartile range.

h MH: minimally invasive hysterectomy.

i LH: laparoscopic hysterectomy.

j OH: open hysterectomy.

k SCC: squamous cell carcinoma.

l AC: adenocarcinoma.

m AS: adenosquamous carcinoma.

n DSI: depth of stromal invasion.

o RFS: recurrence-free survival.

p OS: overall survival.

### Preoperative Diagnostic Performance

Integrated models combining MR images with 5 clinical parameters demonstrated variable performance across 7 ML algorithms (Table 2).

For parametrial invasion diagnosis, classical ML models generally exhibited poor sensitivity (0.00‐0.57). Specifically, RF and KNN failed to detect any positive cases (sensitivity=0.00), while SVM and DT showed low sensitivity (0.57 and 0.50) and specificity (0.56 and 0.59). On the contrary, imbalanced-data variants exhibited improved performance: (1) Balanced RF achieved balanced metrics (sensitivity=0.81, specificity=0.85, F1-score=0.64); (2) weighted KNN attained high sensitivity (0.98), while weighted DT prioritized specificity (0.93).

For lymph node metastasis detection, classical ML models showed limited sensitivity (0.31‐0.66), with RF achieving high specificity (0.87) and SVM delivering the best traditional performance (sensitivity=0.66, specificity=0.52, F1-score=0.54). Imbalanced-data variants exhibited enhancements: (1) Weighted KNN demonstrated optimal overall performance (sensitivity=0.67, specificity +=0.61, F1-score=0.58); (2) balanced RF achieved high specificity (0.87) and precision (0.68); and (3) weighted DT showed marginal improvement over classical DT (sensitivity=0.38 vs 0.53, F1-score=0.42 vs 0.49).

**Table 2.** The result of the preoperative diagnosis using various kinds of integrated ML^a^ models for patients with stage IA1 (LVSI+)^b^ to IIB CC^c^. All models were constructed using both MRI and clinical data.

Models	Sensitivity	Specificity	Accuracy	Precision	F1-score
Parametrial invasions prediction					
Classical ML					
KNN^d^	0.00	0.99	0.83	0.00	0.00
SVM^e^	0.57	0.56	0.56	0.21	0.31
DT^f^	0.50	0.59	0.57	0.20	0.29
RF^g^	0.00	0.99	0.83	0.00	0.00
Unbalanced ML					
Balanced RF	0.81	0.85	0.85	0.53	0.64
Weighted KNN	0.98	0.25	0.37	0.21	0.35
Weighted DT	0.29	0.93	0.82	0.46	0.35
Lymph node metastasis prediction					
Classical ML					
KNN	0.31	0.77	0.60	0.46	0.37
SVM	0.66	0.52	0.57	0.46	0.54
DT	0.53	0.60	0.58	0.45	0.49
RF	0.33	0.87	0.66	0.60	0.42
Unbalanced ML					
Balanced RF	0.45	0.87	0.71	0.68	0.54
Weighted KNN	0.67	0.61	0.63	0.52	0.58
Weighted DT	0.38	0.74	0.60	0.47	0.42

a ML: machine learning.

b LVSI: lymphovascular space invasion.

c CC: cervical cancer.

d KNN: K-nearest neighbors.

e SVM: support vector machine.

f DT: decision tree.

g RF: random forest.

### Postoperative Prediction Performance

**Figure 2.**
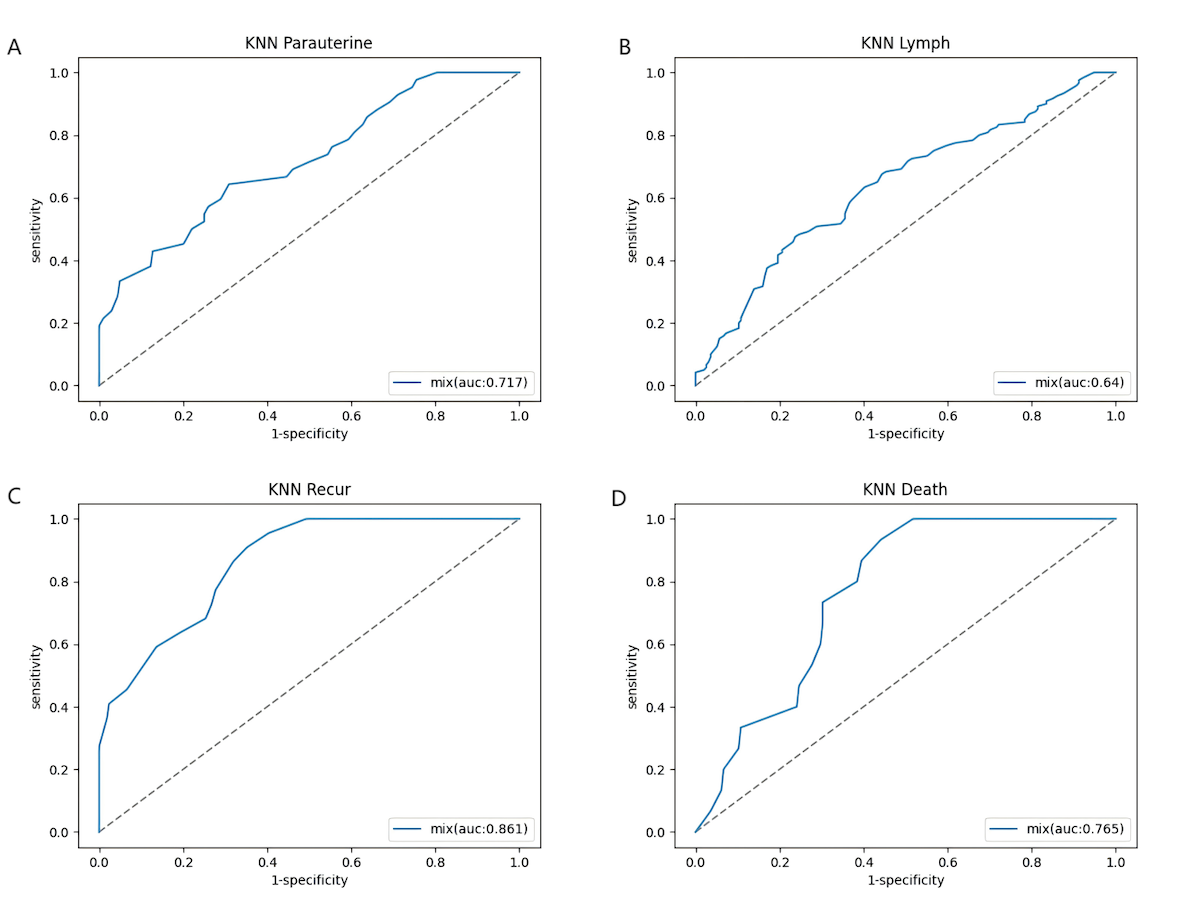
Receiver operating characteristic curves for postoperative recurrence and mortality prediction in patients with International Federation of Gynecology and Obstetrics stage IA1 (lymph-vascular space invasion+) to IIB CC using various integrated weighted K-nearest neighbor (KNN) models. The x-axis represents 1-specificity, and the y-axis represents sensitivity. (A and B) Integrated models incorporating both clinical parameters and magnetic resonance imaging data were used to evaluate parametrial invasion (A) and lymph node metastasis (B). (C and D) Integrated models incorporating both clinical parameters and MRI data were used to predict postoperative recurrence (C) and mortality (D).

For prognostic prediction (Table 3), all classical ML models (KNN, SVM, DT, and RF) failed to identify recurrence or mortality cases (sensitivity=0.00). Imbalanced data variants exhibited divergent outcomes: For recurrence prediction, (1) weighted KNN achieved clinically actionable performance (sensitivit=0.80, specificity=0.96, F1-score=0.73); (2) balanced RF detected recurrences effectively (sensitivity=0.80) but exhibited poor specificity (0.37); and (3) weighted DT failed to identify recurrence cases (sensitivity=0.00). For mortality prediction, (1) weighted KNN showed perfect specificity and precision (specificity=0.99, precision=0.99) but demonstrated low sensitivity (0.33); (2) balanced RF showed limited detection capability (sensitivity=0.33, F1-score=0.11); and (3) weighted DT failed to identify mortality cases (sensitivity=0.00). Among all models, weighted KNN yielded the highest AUC for recurrence (0.861) and mortality (0.765) (Figure 2). Given the importance of sensitivity in guiding postoperative treatment and follow-up, weighted accuracy was introduced as an evaluation metric, and multiple weighting strategies were tested (Table S2 in Multimedia Appendix 1). Results revealed that increasing the weight of sensitivity led to improved detection accuracy for potential recurrence and mortality cases.

For individualized survival time prediction (Table 4), weighted KNN achieved the best performance for recurrence with the lowest MAE (8.53 mo) and highest C-index (0.83), while regression tree (RT) achieved the best performance for mortality (MAE=4.36 mo; C-index=0.99).

**Table 3.** The result of postoperative prognosis prediction using various kinds of integrated ML^a^ models for patients with stage IA1 (LVSI+)^b^ to IIB CC^c^. All models were constructed using both MRI and clinical data.

Models	Sensitivity	Specificity	Accuracy	Precision	F1-score
Recurrence prediction					
Classical ML					
KNN^d^	0.00	0.89	0.89	0.00	0.00
SVM^e^	0.00	0.89	0.89	0.00	0.00
DT^f^	0.00	0.89	0.87	0.00	0.00
RF^g^	0.00	0.89	0.89	0.00	0.00
Unbalanced ML					
Balanced RF	0.80	0.37	0.41	0.12	0.21
Weighted KNN	0.80	0.96	0.94	0.67	0.73
Weighted DT	0.00	0.96	0.86	0.00	0.00
Mortality prediction					
Classical ML					
KNN	0.00	0.92	0.92	0.00	0.00
SVM	0.00	0.92	0.92	0.00	0.00
DT	0.00	0.92	0.92	0.00	0.00
RF	0.00	0.92	0.92	0.00	0.00
Unbalanced ML					
Balanced RF	0.33	0.71	0.69	0.07	0.11
Weighted KNN	0.33	0.99	0.97	0.99	0.50
Weighted DT	0.00	0.96	0.86	0.00	0.00

a ML: machine learning.

b LVSI: lymphovascular space invasion.

c CC: cervical cancer.

d KNN: K-nearest neighbors.

e SVM: support vector machine.

f DT: decision tree.

g RF: random forest.

**Table 4.** The mean absolute error and C-index of postoperative RFS^a^ and OS^b^ prediction using integrated models using MR^c^ and clinical parameters for patients with stage IA1 (LVSI +)^d^ to IIB CC^e^. All models were constructed using both MR images and clinical data.

Models	Mean absolute error (month)	Concordance index
RFS prediction		
Weighted KNN^f^	8.53	0.83
SVM^g^	9.13	0.33
RF^h^	8.83	0.5
RT^i^	10.74	0.17
OS prediction		
Weighted KNN	4.69	0.67
SVM	5.31	0
RF	19.81	1
RT	4.36	1

a RFS: recurrence-free survival.

b OS: overall survival.

c MR: magnetic resonance.

d LVSI: lymphovascular space invasion.

e CC: cervical cancer.

f KNN: K-nearest neighbors.

g SVM: support vector machine.

h RF: random forest.

i RT: regression tree.

### AI-Assisted Contouring and Prognosis Prediction System

**Figure 3.**
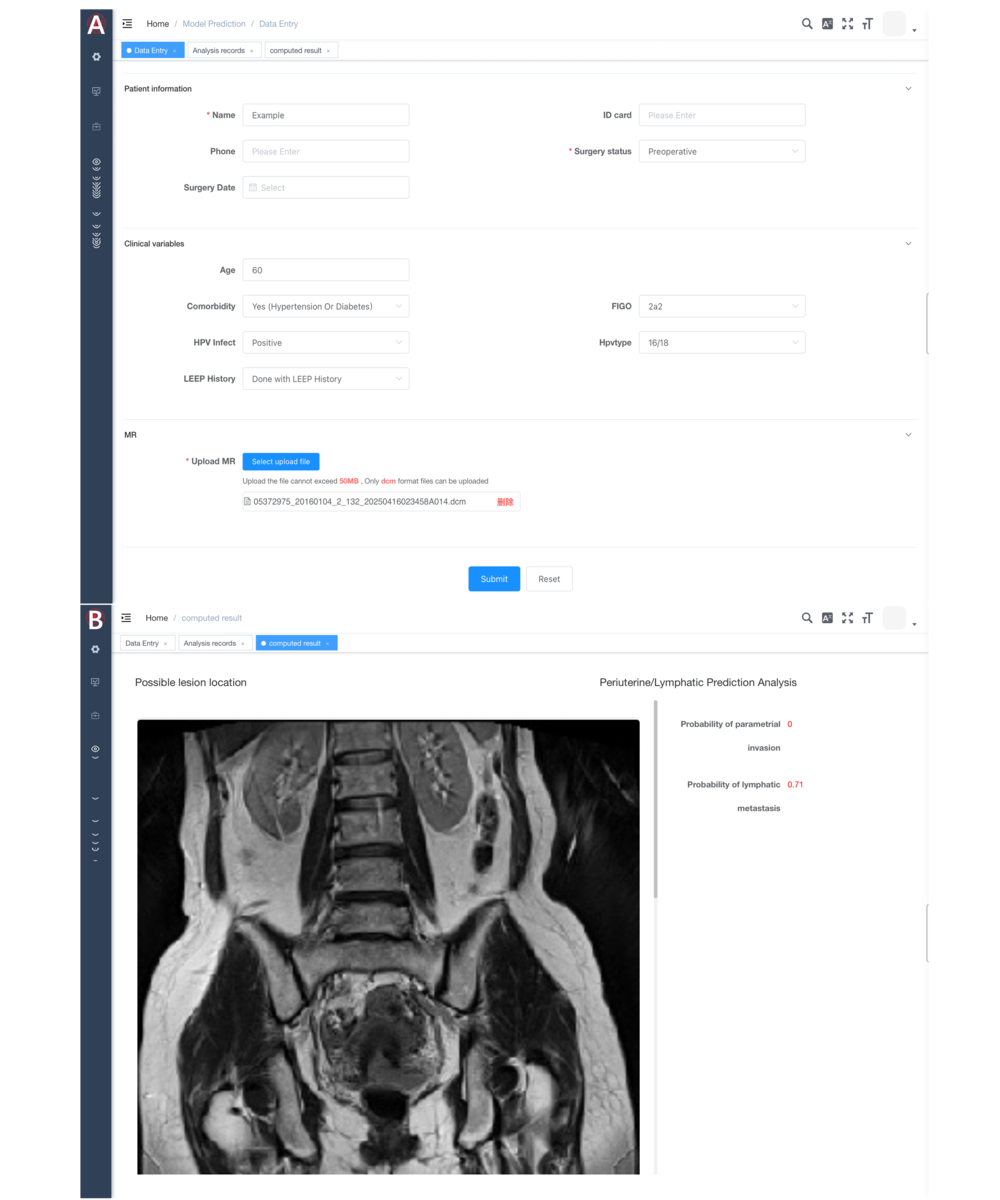
Screenshots of the web-based diagnostic software [27] developed to assist with preoperative evaluation of parametrial invasion and lymph node metastasis, and to predict postoperative recurrence and mortality, including individualized recurrence-free survival (RFS) and overall survival (OS) estimations. (A) By inputting the required clinical parameters and uploading MR images in DICOM format, users initiate analysis through the “submit” function. (B) Following submission, the software evaluates the possible lesion area and generates predictions for parametrial invasion and lymph node metastasis. If the “surgery status” is set to “postoperative,” probabilities of recurrence and mortality as well as specific RFS and OS estimations will be generated.

To enhance the clinical applicability of the prediction model and improve physicians’ access, usability, and integration into practice, the optimal artificial intelligence (AI) prediction models were embedded into a web-based software platform designed to assist in preoperative evaluation and prognosis prediction on MR images (Figure 3) [27]. By inputting key preoperative clinical parameters and uploading DICOM (DCM) files of preoperative MR images, the system can automatically identify possible lesions and generate predictions regarding the patient’s risk of parametrial invasion and lymphatic metastasis. Similarly, by entering relevant postoperative clinical, surgical, and pathological data along with uploading postoperative MR images, the system can estimate the patient’s risk of recurrence and mortality, as well as predict individualized durations of RFS and OS.

## Discussion

### Principal Findings

This study developed integrated ML models combining clinicopathological characteristics with MRI data for preoperative assessment of parametrial invasion and lymph node metastasis, alongside postoperative prognosis prediction in early-stage CC. Our key findings demonstrate the following: (1) Integrated models improved diagnosing sensitivity of parametrial invasion and lymph node metastasis, enhancing preoperative staging accuracy to better guide surgical plan; (2) postoperative prognostic models achieved robust performance in individualized recurrence (AUC 0.861) and survival prediction (AUC 0.765), providing a novel tool for precise adjuvant treatment and individualized follow-up strategy; and (3) a clinically deployable AI platform operationalizes these models for clinical workflow integration, enabling automated risk stratification.

### Comparison to Prior Work

#### Preoperative Assessment

Our integrated models outperformed existing approaches. For parametrial invasion, balanced RF achieved substantially higher sensitivity (0.81) and F1-score (0.64) than a pathology-based clinical model (sensitivity 0.53, F1-score 0.54 calculated) [9], suggesting complete MRI provides more comprehensive prognostic information. While some studies reported better predictive performance for lymph node metastasis using axial T2WI + ADC sequences (sensitivity 0.87, specificity 0.70) [15], this discrepancy likely stems from our use of coronal T2WI versus their multi-planar/functional protocols. Crucially, our integrated models surpass radiologist assessments for both parametrial invasion (sensitivity 0.62‐0.75, specificity 0.84‐0.87) [28,29] and lymph node metastasis (sensitivity 0.54, AUC 0.65) [30], further validating the advantage of integrating clinical and imaging data in preoperative staging and surgical planning [31].

#### Postoperative Prognosis

Weighted KNN’s strong performance (recurrence AUC 0.861; survival AUC 0.765) reflects its unique suitability for our clinical context. Unlike DL architectures requiring massive datasets to realize their potential, this non-parametric method leverages local neighborhood patterns while resisting overfitting, proving particularly effective for limited-sample scenarios where conventional DL advantages remain constrained [32,33]. Critically, this technical alignment serves a fundamental clinical imperative: prognostic prediction prioritizes minimizing missed recurrences (false negatives), where diagnostic harm substantially outweighs false positives, reflecting radiologists’ conservative tendency to avoid overdiagnosis. To operationalize this priority, we implemented weighted accuracy metrics—demonstrating that elevating sensitivity weights enhances high-risk case detection (Table S2 in Multimedia Appendix 1). This strategic focus enables superior identification of positive cases compared to conventional clinical diagnosis, aligning with established ML advantages in sensitivity-driven contexts [8,34]. This multimodal integration resonates with advancements [¹⁸F]Fluorodeoxyglucose (FDG)-positron emission tomography/CT feature integration [35], confirming that combining data sources overcomes single-modality constraints.

### Strengths and Limitations

The core strength of our study lies in developing a comprehensive AI-driven clinical support system that uniquely bridges preoperative staging and postoperative prognosis. By integrating MRI with clinicopathological data through imbalanced-data algorithms (eg, balanced RF and weighted KNN), we significantly enhanced sensitivity for detecting parametrial invasion and lymph node metastasis preoperatively alongside postoperative recurrence and mortality. Crucially, this framework was operationalized through a clinically deployable platform enabling automated DICOM analysis, risk stratification, and individualized survival time prediction, thus forming a closed-loop decision pathway from diagnosis to follow-up.

Despite these advances, several limitations merit careful consideration. Foremost among these is the limited population and tertiary-center recruitment bias. The patient population in this study was limited and may not adequately represent the broader clinical population, particularly individuals in primary care settings or from different geographic regions. Patients were recruited from 2 specialized hospitals, which may have introduced selection bias due to potential differences in disease severity or demographic characteristics. Crucially, only 23.2% of the patients with CC initially identified possessed the necessary MRI data for modeling inclusion. This limitation stemmed primarily from the requirement for locally archived DICOM files. Importantly, most excluded patients underwent initial MRI scans at external institutions—predominantly primary care hospitals where only non-digitalized paper reports were available, precluding DICOM file retrieval. While subtle technical variations in MRI scanners or protocols across institutions may exist, their impact on model generalizability is likely minor compared to the dominant limitation of sample size scarcity. Second, the relatively short follow-up period (<5 y) restricted our ability to evaluate long-term outcomes. This limitation may hinder the detection of delayed disease recurrence and treatment-related effects, especially in chronic disease contexts where complications can take several years to manifest. Third, although DL models are widely recognized for their superior performance when applied to large datasets, this study exhibited suboptimal performance due to the limited sample size and parameter constraints.

### Future Directions

To bridge these gaps and advance clinical translation, 3 strategic priorities emerge. First, we will expand the cohort through multicenter collaborations targeting 1200+ patients—deliberately enriching underrepresented positive cases—while extending follow-up beyond 5 years to capture long-term outcomes. Second, transfer learning will be implemented to develop advanced 3D CNN architectures using volumetric DICOM data, overcoming current sample size barriers and unlocking DL’s latent potential. Third, recognizing that coronal T2WI—while optimal for bilateral tumor assessment—represents only one facet of MRI’s diagnostic capability, we will implement multidimensional sequence analysis. Concurrently, we will systematically integrate axial/sagittal planes and functional sequences (DWI, DCE-MRI) to refine microinvasion detection, ultimately validating this optimized system through prospective trials assessing its impact on surgical planning and survival outcomes.

### Conclusions

In conclusion, this study used ML techniques to develop diagnostic and prognostic models using clinical and MRI data for patients with FIGO stage IA1 (LVSI+) to IIB CC. These models were designed to detect preoperative parametrial invasion and lymph node metastasis and to predict postoperative survival and recurrence. We trained and externally validated the models using data from 250 patients across 2 tertiary hospitals. In both diagnostic and prognostic applications, integrated ML models, particularly weighted KNN, demonstrated favorable performance with clinically applicable sensitivities. These findings suggest that integrated ML models may offer a valuable tool for improving preoperative staging and individualized prognosis prediction in CC, supporting more personalized and precise treatment strategies.

## Supplementary material

10.2196/69057 Multimedia Appendix 1 Model architecture and training details, including modified 3D U-Net structure and optimized Focal Loss implementation.

## Abbreviations

AI: artificial intelligence
AUC: area under the receiver operating characteristic curve
C-index: concordance index
CC: cervical cancer
CT: computed tomography
DL: deep learning
DSI: depth of stromal invasion
DT: decision tree
FIGO: International Federation of Gynecology and Obstetrics
HPV: human papillomavirus
IQR: interquartile range
KNN: K-nearest neighbor
LVSI: lymph-vascular space invasion
MAE: mean absolute error
MCC: Matthews correlation coefficient
mIoU: mean intersection over union
ML: machine learning
MR: magnetic resonance
MRI: MR imaging
NCCN: National Comprehensive Cancer Network
OS: overall survival
PET: positron emission tomography
RF: random forest
RFS: recurrence-free survival
ROI: region of interest
T2WI: T2 weighted imaging
