# Supplementary material for: Machine Learning for Preoperative Assessment and Postoperative Prediction in Cervical Cancer: Multicenter Retrospective Model Integrating MRI and Clinicopathological Data
Source: JMIR Cancer. 2025 Sep 12;11:e69057. doi: 10.2196/69057 (PMC12431160; doi:10.2196/69057)
Supplement: Multimedia Appendix 1 [file cancer-v11-e69057-s001.docx]

# *Supplementary:*

The encoding path included a 1×2×2 max-pooling layer followed by two 3×3×3 convolutional layers, each activated by a ReLU function^[1].^ The decoding path mirrored this structure in reverse. Using skip connections, features from layers of the same resolution in the encoding path were passed directly to the decoding path, thereby preserving the original high-resolution spatial information. The squeeze-and-excitation layer was modified and embedded into the 3D U-Net architecture. The input data, shaped as (C×W×H×D), was first compressed to (C×1×1×1) via global pooling. To capture inter-channel dependencies while reducing computational complexity, the feature dimension was reduced to (C/2×1×1×1), passed through a ReLU activation, and then restored to its original size (C×1×1×1) using a fully connected layer. A normalized weight between 0 and 1 was then obtained through a Sigmoid activation function^[1]^ and applied to reweight the original input channels, which were then reshaped back to (C×W×H×D). Subsequently, the grayscale image was segmented using a Sigmoid-based probability map that classified each voxel as a positive or negative sample. An improved Focal Loss function^[2]^ was introduced to address sample imbalance, where MR image sequences contained significantly more frames without lesion markers than those with them. The Focal Loss applied a weighting factor α to balance positive and negative samples and down-weighted easy examples using a power function. In this study, the optimal parameters were α = 0.25 and γ = 2.

[1] I. Goodfellow, Y. Bengio and A. Courville, Deep learning. 2016: MIT press.

[2] T.-Y. Lin, P. Goyal, R. Girshick, et al. Focal loss for dense object detection. in Proceedings of the IEEE international conference on computer vision. 2017.

**
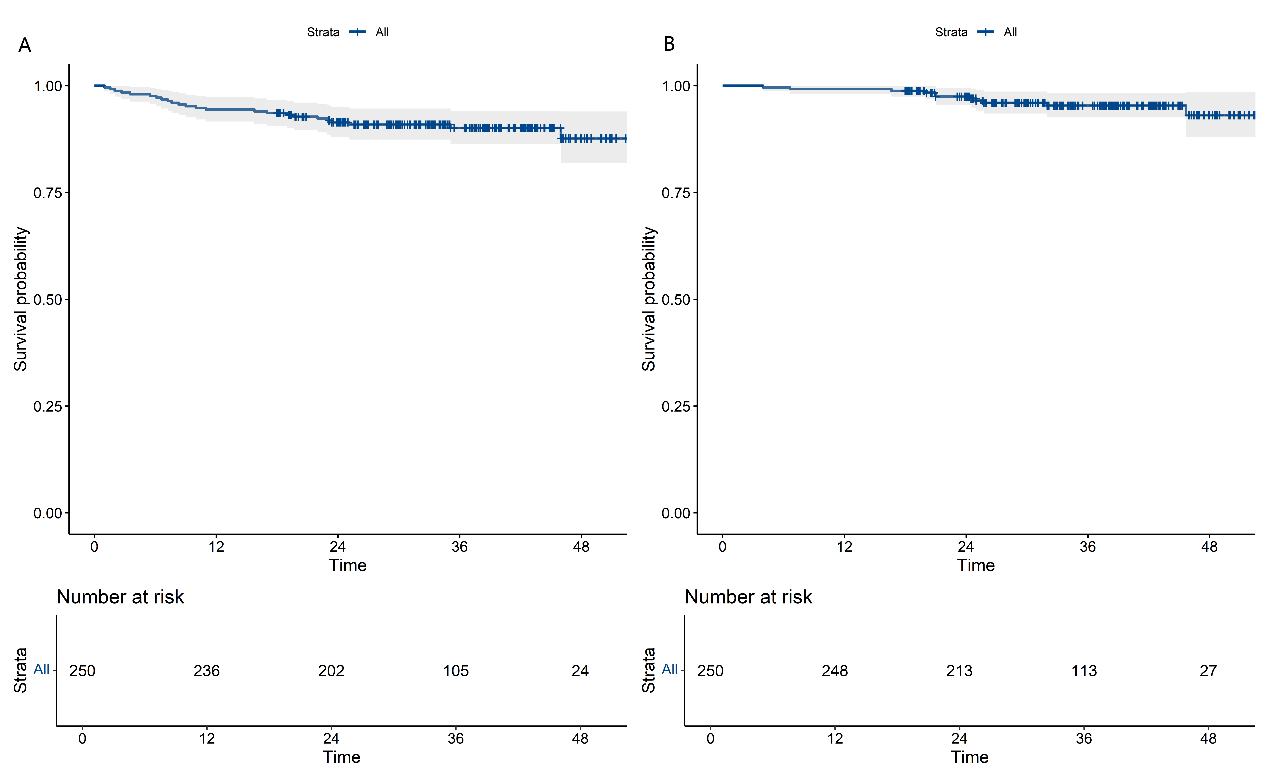
*Figure S1.* Kaplan–Meier curves for 5-year RFS (A) and OS (B) in patients with FIGO stage IA1 (LVSI+) to IIB cervical cancer.**

The table below each curve displays the number of patients at risk at each time point. The shaded light blue area represents the 95% confidence interval for the estimated survival probability.

***Table S1***

**Clinical Parameters Selected for Preoperative and Postoperative ML Prediction Models for Stage IA1 (LVSI +) to IIB Cervical Cancer Patients.**

| Preoperative Clinical Parameters | Postoperative Clinical Parameters |
| --- | --- |
| Age, years | Age, years |
| Comorbidity | FIGO Stage |
| HPV Infection | Comorbidity |
| SCCA, ng/mL | HPV Infection |
| LEEP History | LEEP History |
|  | Post-surgery Adjuvant Therapy |
|  | Surgery Approach |
|  | Operative Time, min |
|  | Blood Loss, ml |
|  | Transfusion |
|  | Tumor Size, cm |
|  | Histology |
|  | DSI |
|  | LVSI |
|  | Surgical Margin |
|  | Parametrial Involvement |
|  | LN Metastasis |
|  | Keratinization |
|  | P53 |
|  | P16 |
|  | Ki67 |

Note:

**Table S2**

**The Result of Postoperative Prognosis Prediction Using Integrated Models Using MR and Clinical Parameters for Stage IA1 (LVSI +) to IIB *Cervical Cancer* Patients.**

|  | Recurrence Prediction | Death Prediction |
| --- | --- | --- |
| **Weighted Accuracy = Sensitivity × 0.3 + Specificity × 0.7** | | |
| Sensitivity | 0.41 | 0.33 |
| Specificity | 0.98 | 0.89 |
| Accuracy | 0.92 | 0.85 |
| Precision | 0.64 | 0.19 |
| F1-Score | 0.50 | 0.24 |
| Weighted Accuracy | 0.81 | 0.72 |
| **Weighted Accuracy = Sensitivity × 0.5 + Specificity × 0.5** | | |
| Sensitivity | 0.91 | 0.93 |
| Specificity | 0.65 | 0.56 |
| Accuracy | 0.67 | 0.59 |
| Precision | 0.21 | 0.14 |
| F1-Score | 0.34 | 0.24 |
| Weighted Accuracy | 0.78 | 0.75 |
| **Weighted Accuracy = Sensitivity × 0.6 + Specificity × 0.4** | | |
| Sensitivity | 0.95 | 0.99 |
| Specificity | 0.60 | 0.48 |
| Accuracy | 0.63 | 0.52 |
| Precision | 0.20 | 0.13 |
| F1-Score | 0.33 | 0.23 |
| Weighted Accuracy | 0.81 | 0.79 |
| **Weighted Accuracy = Sensitivity × 0.7 + Specificity × 0.3** | | |
| Sensitivity | 0.99 | 0.99 |
| Specificity | 0.50 | 0.48 |
| Accuracy | 0.55 | 0.52 |
| Precision | 0.17 | 0.13 |
| F1-Score | 0.30 | 0.23 |
| Weighted Accuracy | 0.85 | 0.85 |

Note: All models were constructed using weighted KNN algorithm with both MRI and clinical data.
